# Measuring researcher independence using bibliometric data: A proposal for a new performance indicator

**DOI:** 10.1371/journal.pone.0202712

**Published:** 2019-03-27

**Authors:** Peter van den Besselaar, Ulf Sandström

**Affiliations:** 1 Network Institute and Department of Organization Sciences, Vrije Universiteit Amsterdam, Amsterdam, The Netherlands; 2 Department of Industrial Economics and Management, KTH Royal Institute of Technology, Stockholm, Sweden; Max Planck Society, GERMANY

## Abstract

Bibliometric indicators are increasingly used to evaluate individual scientists–as is exemplified by the popularity of the many other publication and citation-based indicators used in evaluation. These indicators, however, cover at best some of the quality dimensions relevant for assessing a researcher: productivity and impact. At the same time, research quality has more dimensions than productivity and impact alone. As current bibliometric indicators are not covering various important quality dimensions, we here contribute to developing better indicators for those quality dimensions not yet addressed. One of the quality dimensions lacking valid indicators is an individual researcher’s *independence*. We propose indicators to measure different aspects of independence: two assessing whether a researcher has developed an own collaboration network and two others assessing the level of thematic independence. Taken together they form an *independence indicator*. We illustrate how these indicators distinguish between researchers that are equally productive and have a considerable impact. The independence indicator is a step forward in evaluating individual scholarly quality.

## Introduction

The use of (bibliometric) indicators for evaluating research performance has become rather common, at the level of research organizations and research teams, but after the development of the H-index also at the individual level. The number of indicators has been growing, and a paper published a few years ago already counted 108 bibliometric indicators at the individual level [1]. Despite the large number, almost all of these indicators are focusing on productivity (publications) and impact (received citations). So, these measure in many different ways the same quality dimensions, as is visible in recent work on individual level metrics [2, 3, 4, 5]. Only a few of the 108 indicators are focusing on another quality dimension, namely of the co-author network. Other reviews of individual-level bibliometric indicators confirm this picture. In a study on the relations between bibliometric indicators, Bollen and colleagues did a principal component analysis of 38 citation and usage indicators [6]. The types of indicators included are citation-based, but also indicators derived from download data (click stream data). The principal component analysis indicated two dimensions of impact: (i) short term impact (usage) versus long-term impact (citations), and (ii) popularity versus prestige. Many indicators focus on scientific impact of papers and related on the impact of journals, and only a few network-type indicators have been developed, using the co-author network to measure an author’s impact [1, 7].

In parallel, a discussion has emerged amongst evaluators and bibliometricians about the reliability and validity of the indicators, and increasingly criticism is formulated on the indicators and their use [8, 9, 10]. The critique can be summarized as follows: (i) peer review should remain the main procedure, and bibliometric indicators can at best inform peer review; (ii) the quality of indicators needs permanent scrutiny; (iii) evaluation should relate to goals; and (iv) indicators have perverse effects. Moreover, as bibliometric data have become available widely, ‘everybody’ is able to calculate indicators, which is seen as very risky, as most non-professionals do not understand the meaning of the indicators, and apart from that, are not able to calculate the indicators in a correct, e.g. field-normalized, way [11].

The criticism on bibliometric indicators neglects the many problems with peer review, which we do not discuss here [12], and it focuses strongly on the so-called perverse effects of indicators: Researchers may start maximizing their indicator scores; instead of optimizing their real academic quality and impact. Yet, the often-claimed perverse effect of indicators (counting publications for example) lacks empirical support [13, 14].

In this paper, we focus on the other main point, the validity of the indicators: Do bibliometric indicators measure what we want to measure? Most bibliometric indicators focus on productivity, impact, and quality of the collaboration network. Over the years, many studies have indicated that bibliometric indicators do well as predictors of peer review in grant decisions [15, 16]. However, that would at the same time point at another problem, as the currently available indicators do not cover some important dimensions of a more inclusive measurement of scholarly quality–which should be the basis of grant or job decisions. Our aim here is to enrich the set of indicators, which should help to measure scientific quality in a comprehensive way.

What constitutes a high-quality researcher [17]? The metrics discussion has in fact been rather restricted and misses the link to the larger evaluation frameworks that are or might be used, on the institutional and team level, or related to grant and paper evaluation. Here we focus on assessing researchers for hiring and tenure and promotion [18], and leave other evaluation contexts out, such as evaluating the organization of research, research funding, and research policy [19].

In order to uncover what counts as quality in this context, one may interview those involved in setting the standards: active researchers [19]. A previous study interviewed some 40 panel members, who mentioned a large and diverse set of different quality dimensions they used in evaluation of grant proposals, and in evaluation for job applications [20]. Several dimensions could be distinguished: (i) Productivity proved to be the main criterion, as were earlier grants, originality, a broad scope, and independence; (ii) other work-oriented personal characteristics were mentioned too–like commitment and hard-working, and ambition, and when it is about academic jobs, also (iii) social characteristics–like being a pleasant person, and a team player (Table 1).

**Table 1:** Evaluation dimensions.

JOB EVALUATION	GRANT EVALUATION
Publication record	Publication record
Earlier acquired grants	Well-developed proposal
Proactive	Hot research topic
Independence	Originality
Writing skills	General comprehensiveness
Ability to work hard	International experience
Perseverance	Self-consciousness
Ambition	Ambition
Being social	Authenticity
Enthusiasm	Enthusiasm

One of the criteria frequently mentioned for jobs is independence. As science develops through original and new ideas, there is a need for independent researchers that challenge the existing common views and open up new lines of research. Independence, in this sense, means breaking with existing lines of work and trying to come with something new. To achieve higher academic positions, or to get prestigious grants, one should have shown this type of independence.

The question of independence gets an additional relevance through the growth of research collaboration and team science [21, 22, 23]. If most work is collaborative, this must have implications for the assessment processes and the indicators deployed. An independence indicator may be one of the instruments to disentangle the performance from complex network dependencies. It helps to fill the gap in the existing set of quantitative indicators [18]. While focusing on independence, we are aware that more quality dimensions are used–as shown in Table 1 –such as reputation (measured e.g., by the number of grants, prizes won, invited lectures at conferences), international mobility (stays abroad), or societal impact (third-party funding). Also for these quality dimensions, indicators need further development, and appropriate data need to be collected. For example, *earlier grants* are often mentioned as an indicator of scholarly quality. However, it is not so clear how to add up grants to an indicator value, and how to weight the various aspects, such as number of grants, total amount of funding, the prestigious grants, and the same holds for operationalizing ‘stays abroad’ in order to measure mobility [24].

## Independence

What is meant with independence? We briefly review the policy discussion about independence and then continue with a more systematic approach. Not only do peers—in interviews mentioned above–point at independence, also science organizations like the US National Academies of Science (NAS) emphasize the importance of independence, and warned that the conditions for becoming independent were deteriorating [25] (see also [26]). NAS claimed that the age at which researchers in the US got the first independent grant is increasing, suggesting that independence is being able to follow one’s own research interests and develop own research lines, and having the resources to do so. Getting own grants at a later age would imply that researchers are becoming independent too late, something that also may work negatively on attracting new generations to scientific research. As the NAS committee found that requests for grants from new generation investigators most often are evaluated on the basis of earlier “preliminary results”, most funded research became constrained to well-worn research paths—those previously pursued by the new investigators when they were postdoctoral fellows in established laboratories. In short, innovation was the victim of a system that had become much too risk adverse:

“Furthermore, the study sections want significant assurance that the project will work, and so they require the principal investigator to have surmounted at least some of the experimental hurdles inherent in the project prior to funding. Reasonable young principal investigators are quick to get the message: stay within the confines of known systems and proven technologies, and do not challenge existing beliefs and practices.” [25]

In other words, there is a serious concern that new investigators are being driven to pursue more conservative research projects instead of the high-risk, high-reward research that more likely advance science significantly. The special creativity that younger scientists may bring in may also get lost when they are forced to focus on established research. An ‘independent investigator’ can be defined as one who enjoys independence of thought—the freedom to define the problem of interest and to choose or develop the best strategies and approaches to address that problem–especially during the earlier career phases. Under this definition, an independent scientist may work alone, as the intellectual leader of a research group, or as a member of a consortium of investigators each contributing distinct expertise. Independence does not mean necessarily ‘isolated’ or ‘solitary’, or imply ‘self-sustaining’ or ‘separately funded’ [25].

A good example is the Starting Grants scheme of the ERC [27, 28]. The ERC instructs its panels to select excellent researchers and groundbreaking projects, and one of the few concrete criteria they mention is ‘independence’, defined as having one (or a few) publications without the former PhD supervisor being co-author. The idea is clear, but this indicator is easy to play: If supervisors know this, they may avoid being co-author on all papers. Indeed, in CV analysis we found that applicants list papers without the supervisor, but which in cases may include a co-supervisor. When interviewing the ERC panelists, independence indeed proved a major issue, but how it can be measured remained unclear. Some would argue that single authored articles indicate independence, but in a world where one can hardly do research alone, this becomes a slightly irrelevant indicator. Other indicators mentioned are: being PI on a grant; being lab director or research leader; evaluations of teaching for being sole teacher; letters from research directors about your contribution to a research project (including assigning you a percentage of effort).

That current (bibliometric) indicators have limitations in measuring independence has been pointed out by many [29, 30]. It was also the focus for a working party of the Swedish Research Council for Medicine and Health that was reported in 2007:

“The assessment of independence is a frequently discussed issue, especially when it comes to researchers in large collaborative projects and/or young researchers. (…) Given that research today often includes networks and large collaborative research teams, it may be difficult to discern the independence of individual researchers and especially of the young. A young researcher in a network often shows many publications, but without a prominent place in the author list, which complicates the assessment of the degree of independence.” [31] (our translation)

The importance of independence in the Swedish setting is also illustrated by the longstanding work of docent promotion committees at faculties of medicine. For several years a working group of these committees has been trying to operationalize the rules. One example how they treat independence is the Karolinska Institute’s regulations for those committees:

“Independence can be documented, for example, when the applicant has been obviously senior representative of recent contributions to research, the latter not seldom exemplified by the recent scientific publications, and has conducted his (sic!) own consistent research line after the dissertation. Independence can also be shown through research responsibilities as a supervisor for a PhD student or a guest researcher.” (Instructions for *docent position* at Karolinska Institute, established by the Vice-chancellor March 18, 2014. The Swedish rank ‘docent’ is equal to the US rank “associate professor”)

In the discussions summarized above, independence is mentioned as an important quality of *early career researchers*, with a variety of aspects: (i) Not remaining too close to the research interests of the PhD supervisor(s); (ii) Having an own collaboration network. Independence (for early career researchers) should be distinguished from other—related–quality dimensions (mentioned in Table 1). For example, the originality is an important dimension, but original work can also be done in a team, where the early career researcher is a dependent member. The same holds for working on hot topics. Finally, being successful in grant applications is important, but if one only gets grants because of signs of independence then it should be treated as a different quality dimension.

## Independence indicators

If independence is an important evaluation dimension, the question is whether we can develop indicators to measure independence in a reliable and valid way. This is important, as research shows that without clear indicators, the criteria are often applied differently for different applicants. For example, when female researchers are evaluated they are often considered as ‘dependent’ on their supervisor while male applicants more often were considered as ‘independent’, even when they followed in the research lines of their supervisor [32]. A recent qualitative study performed in Sweden found the same pattern [33]. Results were based on direct observation of committees inside the Swedish Research Council. Therefore, indicators could be used to improve selection processes, and to avoid selectively dropping of criteria and to avoid bias. The policy discussions about independence [25, 31] show in our view that there is a need for a valid and unbiased indicator that could be used to measure independence, reflecting the meaning of the concept and getting it uncoupled from the many gender biased and other stereotypes.

First of all, a good researcher, of course, needs to have acquired excellent research skills and produced relevant results, which can be measured using publications and citations. However, as most research is teamwork, these publications and citations could have been ‘borrowed’ from an excellent team, in which a researcher not necessarily has had the leading role. Therefore, it is important that an early career researcher should also have developed independence. One needs to be able to formulate one of preferably several own independent and promising research lines. This would result in papers on topics that are different from those of the environment where the early career was spent, and with other co-authors than supervisors and colleagues from that period.

Independence is not the only relevant dimension, as Table 1 indicates. One may also think of *originality*, *risk-taking* or *interdisciplinary* as important features of the research lines to be developed, and the *ability to do groundbreaking research*. [28, 34]. Whether the research lines are promising should also be measured: with traditional indicators like top-cited papers, but also using science maps based indicators that position the research lines within the larger landscape: is this a line within a hot or cold field? Is attention for the field growing and if so, fast? Sandström & Sandström [2] and Wang [35] are but two examples of methods for finding hot areas that are based on similarities between papers. This would lead to several other relevant new indicators, which we will not develop here, but briefly mention them as part of our wish list for better indicators [34, 36, 37].

The aim of this paper is to develop an operational concept of independence and to provide a proof of concept. We will define the indicators and show how independence can be measured using data about the co-author network and the publications network. An independent researcher, in the line of the discussion above, has taken the freedom to define the problem(s) he/she want to address and to choose the best strategies and approaches to address that problem(s). Independence in this definition is something with a time dimension: independence emerges over the years. It implies in the first place that a young researcher becomes independent from the environment where he/she was educated. As PhD student, and as Postdoc, a researcher works in projects generally designed by others, often the PI who also functions as a supervisor. After having done that for a while, the researcher should have become skilled and experienced to formulate his/her own research questions and projects at challenging research fronts, unguided by the former supervisors. This, of course, does not mean that independent researchers avoid collaboration, as almost all research nowadays is collaborative. In fact, building up relevant collaboration networks is one of the resources needed for excellent research. The challenge is to formulate indicators that can tease out the level of independence from a network of dependencies.

### The structure of the co-author network

If independence means that a researcher does not depend anymore on the environment where he/she was trained as a researcher, we would expect to see that in the collaboration network. An independent researcher is expected to collaborate with a variety of others, but less so with the former supervisor(s). Therefore, independence can be defined as the quality of the co-author network of the researcher. The size of the network indicates how the environment of a researcher perceives his or her contribution. The more someone has to contribute, the more other researchers want to collaborate, so the more co-authors someone has. A young researcher is often introduced in the academic world through the supervisor. At the beginning of the career, the co-author network of a young researcher is therefore expected to be embedded in the network of the supervisor; something that may be helpful in the first career steps. But, after a while, the co-author network of an independent researcher will significantly differ from the supervisors’ co-author network. This can be measured using two network properties of the co-author *ego-network* of the researcher: (i) the *eigenvector centrality* of the former supervisor in the co-author ego-network of the researcher, and (ii) the *clustering coefficient* of the former supervisor in the ego-network of the researcher.

The *eigenvector centrality* expresses influence in the network: in contrast to the degree centrality, the eigenvector centrality takes into account the connectedness of other nodes. A node is important (the eigenvector centrality is higher) if it is connected to other important nodes. So even if the supervisor is not connected to all nodes in the researcher’s network, if he/she is connected to the important (highly connected) nodes, the supervisor’s eigenvector centrality is still high. The *clustering coefficient* measures the extent to which a node is part of a clique in the larger network. The more a supervisor is part of a specific clique within the researcher’s network, the less the researcher’s network coincides with the network of the supervisor, suggesting higher independence.

The researcher is the center of his/her own ego-network, and will, therefore, have an eigenvector centrality of 1. The more independent a researcher is, the more distinct co-author cliques he/she will be part of, and that is expressed in a low (down to 0) clustering coefficient. If the two network scores of the supervisor are similar to those of the researcher, independence is low; the more the researcher’s network is his/her own, the lower the eigenvector centrality (approaching 0) and the higher the clustering coefficient (approaching 1) of the former supervisor will be.

The proposed indicator may not work in all research fields. If there is only one co-author clique (clustering coefficient = 1), it would be impossible to assess the individual independent contribution of the researcher under evaluation. This may be the case in research fronts where ‘everyone’ is on all papers (hyper-authors) as in some physics subfields. Also in fields where single-authored papers are the dominant mode, the indicator would not work, as co-author networks do not exist. However, in all fields, the share of co-authored papers grows fast [38].

### The cognitive network of the researcher

Being independent also means that the researcher starts to explore new topics and to develop own research lines, by following his or her own ideas. The researcher should move to new research questions not belonging to the research agenda of the former supervisor. This explains why the number of citations and publications may not be decisive in assessing the performance of researchers. The real issue is whether one publishes and is cited because of one’s own good research, and not because of the good performance history of the supervisor. This implies that after a while, the publications of the independent early career researcher should be outside the research front(s) where the former supervisors are or have been active.

To measure whether the researcher developed own research lines, independent from the former supervisor, we downloaded from the Web of Science (Online version) the papers of the researcher and the former supervisor, including their co-authored publications. We selected all papers published until two years after the main career decision: appointment as tenured (associate) professor versus leave academia. The additional two years are included to account for papers that were written and possibly even accepted, but not yet published before promotion/leaving dates. For the supervisor we also include papers in the decade before the PhD project of the researcher in order to have the earlier research lines of the supervisor included in the analysis. In order to account for research lines, we use only the following document types: Articles, Letters, Proceeding Papers and Notes. Reviews are, in our understanding, not representations of an individual researcher’s research line as there are many references to research that might be remote to the researcher in question.

We created the joint paper network of researcher and supervisor using bibliographic coupling. This results in a network of several components and clusters representing different strands of research, which can be used to answer the question if an own research line of the researcher is visible in the network. Or are the own papers of the researcher included in one of the clusters of the supervisors’ papers? If the latter is the case, the researcher has remained within the research program of the supervisor, and no own program was developed. For the joint network, we calculate the similarity between papers as bibliographic coupling. Bibliographic coupling is a measure of similarity between papers in terms of shared cited references, and the number of shared cited references is normalized in relation to the total number of cited references in the two papers. We use Salton’s Cosine Index which varies between 0 and 1 [39]. The bibliographic coupling between two papers, based on Salton’s Cosine Index, is defined as

$$\frac{F_{ij}}{\sqrt{S_{i}S_{j}}}$$

where *F*_*ij*_ is the number of common references of paper *i* and paper *j*, and *S*_*i*_ and *S*_*j*_ is the number of cited references in paper i and *j*, respectively.

Using the similarity between papers, one can break down the set of papers into topical clusters. Many algorithms are available, and here we use the SLM algorithm for this [40]. The topic mapping depends on the choice of a resolution level, and higher resolution levels will create more clusters. In cases where the supervisor(s) have a high number of papers compared to the early career researcher, the higher resolution level will especially lead to more joint clusters, and therefore to a lower share of own topics of the early career researcher. Waltman and Van Eck [40] suggest that for normal community detection with the SLM algorithm, the resolution should be set to 1.0. We tested the clustering for three levels of granularity (0.5; 1.0; 1.5) and this hardly made a difference for the topics-indicator (Table 2). In some cases, the values are slightly different, but the rank order remains the same. We used the resolution value 1.5 for this study.

**Table 2:** The share of own topics by resolution level (granularity).

		Level of resolution	
Researcher	0.5	1.0	1.5
RA	0.50	0.50	0.43
RB	0.25	0.25	0.14
RC	0.50	0.50	0.50
RD	0.00	0.00	0.00

Some of the resulting clusters contain only papers of the supervisor, others only papers of the researcher and again other clusters contain papers of both, as well as co-authored papers. If a researcher has developed her own research line, which would imply that the researcher explores other/new questions, and refers to different literatures, then the similarity will be lower; if he/she continues within the research line of the supervisor, the similarity measure will be higher.

We calculate this independence indicator in the following way: we divide the number of all topical clusters where the researcher is active but the supervisor(s) is not by the sum of these own clusters and the joint clusters of the researcher and the supervisor(s). The indicator value is between 0 (if the researcher has only been active in topics in which the supervisor also has been active at some moment) and 1 (if the supervisor is not visible in any of the researchers’ topical clusters).

### Some complexities

1. Often there is more than one PhD supervisor, and then all of them should be considered in the calculation of the indicators.
2. One would assume that early career researchers are dependent on the supervisor and that later on the researcher starts to do things independently of the former supervisor. However, occasionally the supervisor may follow the former PhD student in these new topics, and in those cases having a shared research line would indicate that the supervisor has become dependent on the former PhD student. The latter pattern would imply that the researcher starts in a new topic and the supervisor moving in later on. In order to distinguish these two different dependence situations, the analysis includes also the publications of the supervisor over the 10 years before the PhD project of the early career researcher. Then we can test whether topics were already occupied by the supervisor or not.
3. It may be the case that the early career researcher has an own topic, in which the former supervisor has been *marginally* active long before the researcher entered these topics. Then we do not consider this as a shared topic because of the time difference.
4. The STEM fields are mainly producing co-authored papers. In the social sciences and humanities this is increasing, but here single-authored papers are still rather frequent. For those non-collaborative research fields, we may have to develop a different type of independence indicator, which is future work.
5. Topic clustering is based on referencing behavior of researchers. This type of analysis gives an indication of independence based on the use of references for building clusters, potential users of the methodology are to be aware of that in cases the data set is small the methods do not apply, and other complementary methods have to be developed.

### Calculation of the overall independence indicator

Above, we have defined four 4 measures related to independence:

I_1:_ The eigenvector centrality of the supervisor in the researcher’s co-author network;
I_2_ The clustering coefficient of the supervisor in the researcher’s co-author network;
I_3:_ The share of own papers of the researcher P_ns_/P_all_ where P_ns_ is the number of publications of the researcher, not coauthored with the former supervisor(s) and P_all_ is the total number of publications of the researcher; and
I_4:_ The share of own research topics of the researcher T_ns_/T_all_ where T_ns_ is the number of research topics of the researcher in which the former supervisor(s) is not active, and T_all_ is the total number of topics of the researcher.

After calculating these four indicators, they can be aggregated into a single score. The researcher’s independence indicator RII is calculated as follows:

$$RII=average\;\left(\left(1-I_{1}\right)+I_{2}+I_{3}+2\;*\;I_{4}\right)/4.$$

Where I_1_ = eigenvector centrality, I_2_ = clustering coefficient, I_4_ = share own papers, and I_4_ = share own topics. We use (1 –I_1_) as the lower values for eigenvector centrality represent higher independence, whereas for the other indicators it is the opposite. I_4_ is weighted double, as the range of the ‘own topics’ scores is between 0 and 0.5 whereas the other indicators I_1_, I_2_, and I_3_ are in a range between 0 and 1.

## Data

This paper intends to be *a proof of concept*, and we apply the indicators on four researchers and their supervisors to show how it works. Measuring independence using these indicators can be done for any researcher given that the person in question has publications covered in citation indexes, e.g. Web of Science. However, we are interested in the question whether the indicator is valid, that is whether it measures independence as taken into account by e.g., panels that select applications for positions or jobs. In order to do that one needs a set of researchers which are similar in various dimensions (age, field, talent, academic performance in terms of publications and citations, etc.) but differ in independence. With such data, we can give a tentative answer to the question whether the measured independence relates to e.g., career success.

For a study on research careers [41], research managers of several universities were asked for names of researchers who aimed for an academic career and who were seen as very talented and promising at the beginning of their careers. These researchers were interviewed in order to find factors leading to career success: personal situation and biography, social support, having a mentor at work, and university career and HRM policies. Additionally, data were collected about the labor market situation in the discipline, and about the interviewees’ publication and citation performance. An important result from that study is that none of the factors correlated with career success–it seemed more the number of factors that worked positively: “being at the right place at the right moment”.

For the current study, we use four researchers from that sample. Researchers RA and RB are from the same STEM specialty, as are researchers RC and RD. RA and RC became full professor, and researchers RB and RD left the university and took a position in industry. For all researchers, the decisive career moment was in their late thirties, some nine years after receiving their PhD degree. To calculate the indicators, we downloaded the bibliometric data (WoS) of the publications of the researchers, as well as those of their PhD supervisors (indicated as SA1 and SA2, SB, SC, and SD1 and SD2). We cleaned the data manually. The data cover the career from the start of the PhD research up to their late thirties. For the supervisor(s) we also include publication data of a decade before the start of the PhD trajectory of the researcher, in order to cover the relevant research portfolio of the supervisors.

## Findings

**Fig 1:**
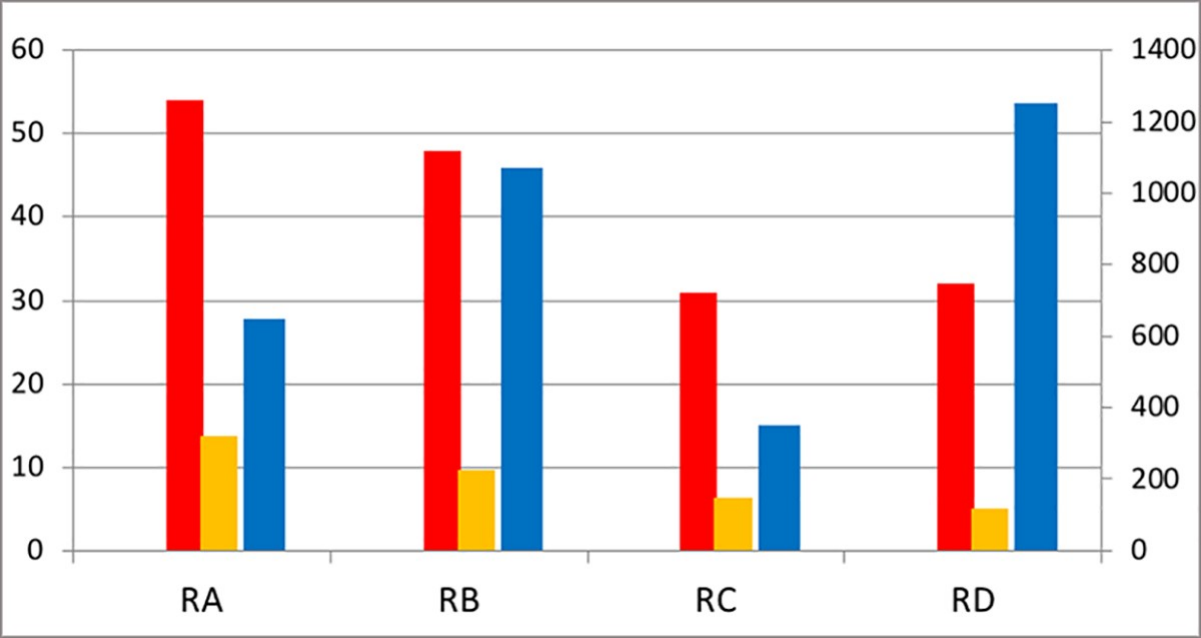
Performance scores of the four researchers at decisive career moment. Number of publications: red (full), orange (fractional) = left axis; number of citations: blue = right axis. Source: WoS The early career of the researchers started in the mid-1990s, and therefore we do not have the data for calculating field normalized (size dependent and size independent) indicators such as visibility in the top 10% cited papers. As the researchers work in similar fields, this is for the current analysis not a problem. Publications and citations were calculated using the ‘create citation report’ function in WoS, which enables to select the papers for the period studied, and the citations received for those publications over that period.

The four researchers all had a good performance level (Fig 1). If publications and citations would be decisive, one would expect RB to have a better academic career than RA, as RB had a comparable output but a much higher impact. The same holds for RD compared to RC.

We first compare RA and RB. One of the researchers had a successful academic career (RA with supervisors SA1 and SA2) and became full professor. The other researcher (RB with supervisor SB) left the university for an industrial R&D lab–so productivity and impact were not decisive here (Fig 1).

All researchers co-authored with their supervisor. In case of RA, about 20% of his publications are co-authored with his two supervisors, and for RB this is 95%, all in the period under. So, some researchers collaborated much more intensively with the supervisor than others did. These different collaboration levels result in rather different network scores: the researchers have in their own ego network by definition an *eigenvector centrality* of 1. Within the ego-network of RB, SB has a high eigenvector centrality (0.91), indicating that SB is almost as central in the network of RB as RB herself. In contrast, the eigenvector centrality of SA1 in the ego-network of RA is very low (0.09), indicating SA1’s (and similarly SA2’s) marginal position in the network of RA. In contrast, the *clustering coefficient* of SB is low (0.11), as low as RB’s clustering coefficient (0.09), but the clustering coefficient of SA1 is high (0.43), very different from the clustering coefficient of RA (0.07). This indicates that SA1 (and SA2) is connected to a specific subset of nodes only. Consequently, we may conclude that RB hardly has an own network, whereas RA does have one.

The other two indicators measure the difference in research lines (topics) between the researcher and the former supervisor. Research lines are analyzed by creating a network of papers, based on bibliographic coupling. Papers that refer to similar literature are in this way clustered. One may calculate the similarity between two oeuvres as the average of the (cosine based) similarity between papers from the oeuvres–which is explained in the methods section. Bibliographic coupling shows that the research lines of RA and SA1/SA2 differ, whereas the research lines of RB and SB are very similar. Visualization of the paper networks illustrates these findings.

**Fig 2:**
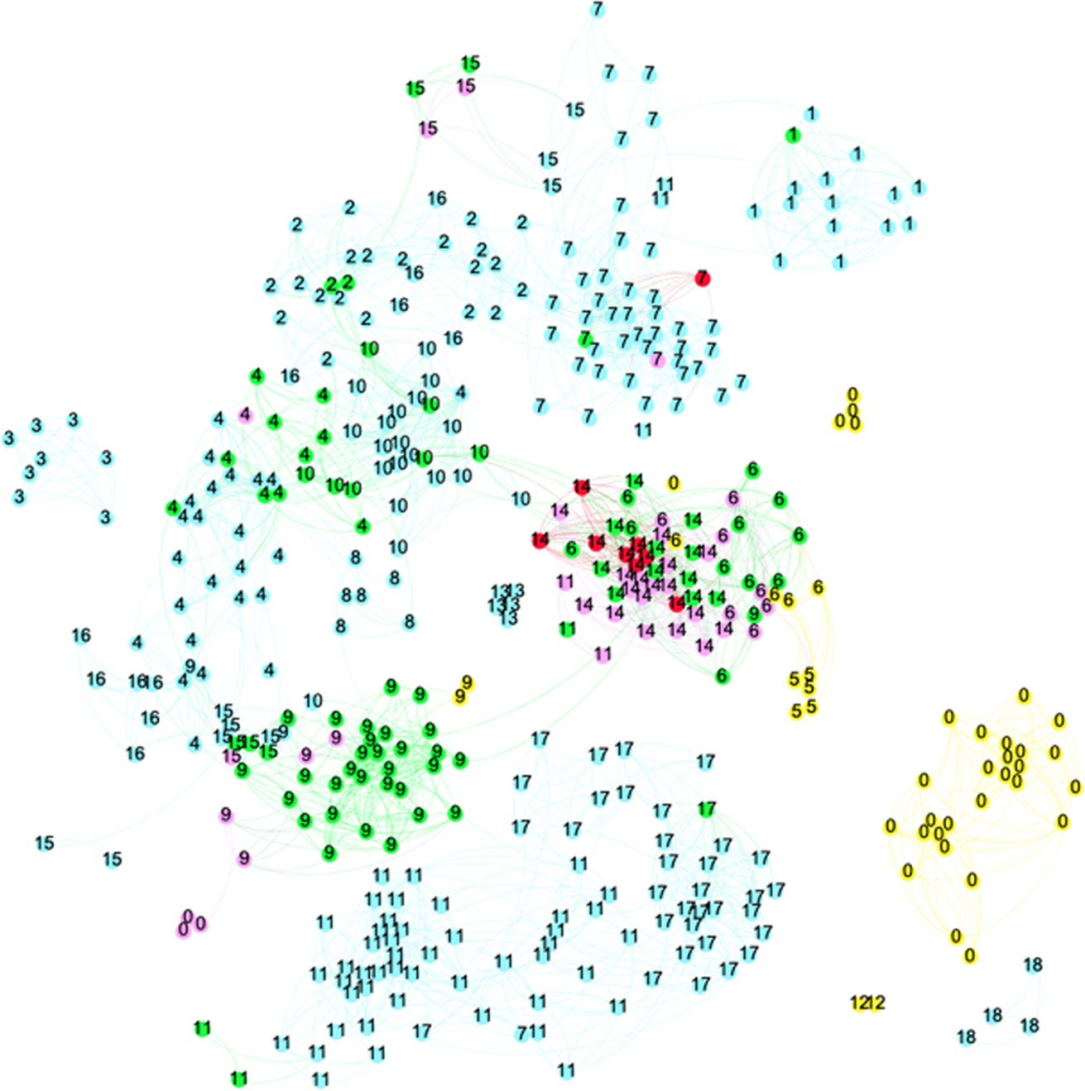
The topic network of researcher RA and two supervisors. Blue/ purple = supervisor(s); Green = coauthored between the two supervisors; Red = researcher co-authored with supervisor(s); Yellow = researcher Numbers indicate the topical clusters.

Fig 2 shows the topics network of the researcher RA and the supervisors. We number the papers by topical cluster, so one may see the variety of topics worked on. The color of the nodes reflects authorship. The light blue nodes are supervisor SA1, the purple nodes are supervisor SA2, and the green nodes are co-authored by the supervisors. The yellow nodes are authored by the researcher, and the red by the researchers plus the supervisor(s). Of course, in all cases, other co-authors may be involved.

Researcher RA worked intensively with supervisor 2 in topic T14 (middle of the map). Other joint clusters but with much fewer papers of the researcher are T6 and T9. But after receiving the PhD degree, RA started to work on other topics, in which neither of the supervisors has been active: T0, T5, and T12. Researcher RA has 83% of the papers and 43% of research topics without the participation of the supervisors. To investigate the further development of RA, we also created a bibliographic coupling map of the papers of RA and SA for another ten-year period (not included here). RA remains detached from his former supervisor(s), and his own research lines have clearly developed extended over that period, unconnected to the work of former supervisors.

**Fig 3:**
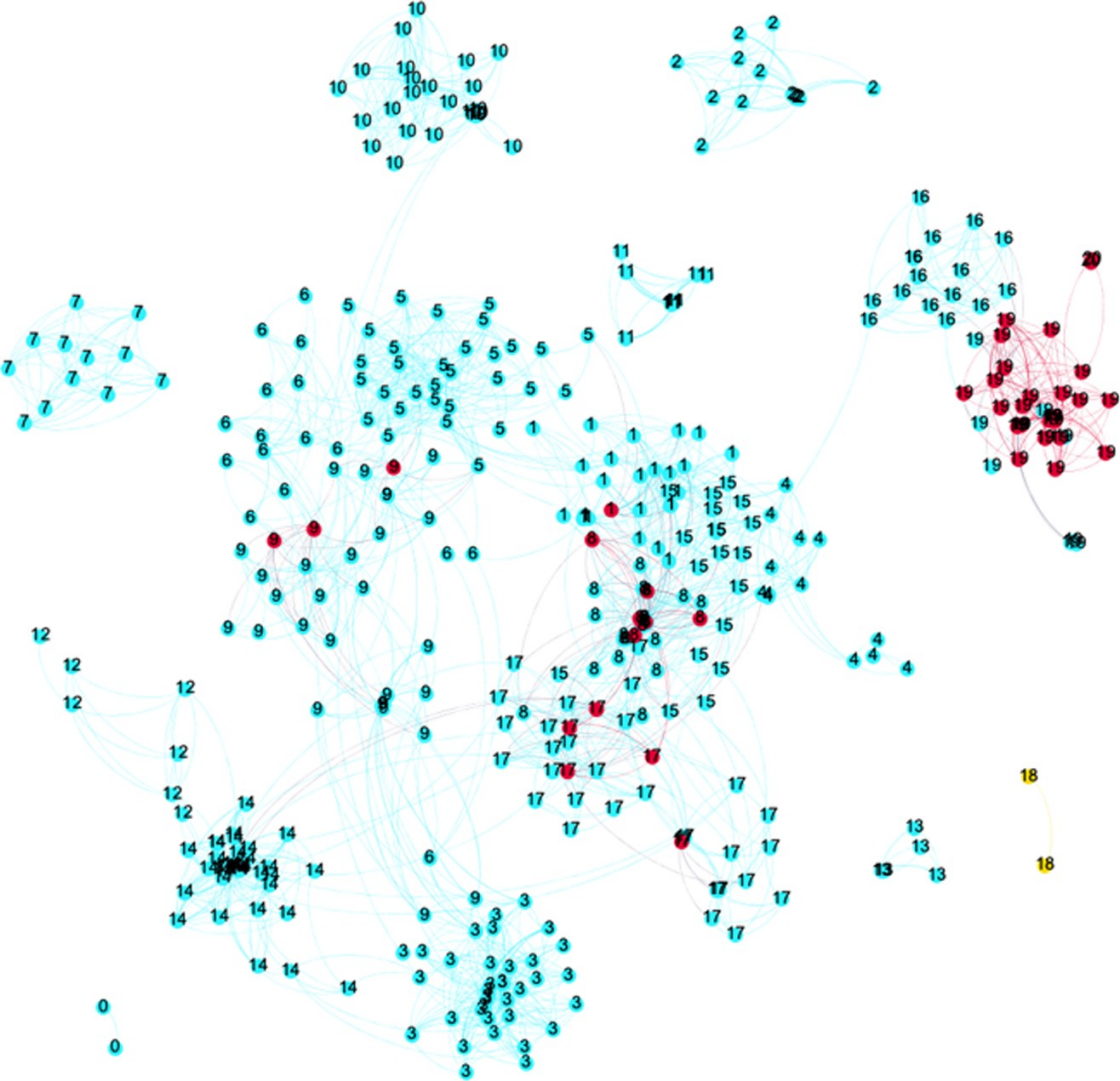
The topic network of researcher RB and supervisor. Blue = supervisor; Red = researcher co-authored with supervisor; Yellow = researcher Numbers indicate the topical clusters.

Fig 3 shows similar information for RB and SB. Only two papers without the supervisor as co-author are visible. These papers also form the only own topic of researcher RB (T18, yellow nodes). All other papers of RB are co-authored with the former supervisor. Cluster T19 includes most of those, and the others are strongly connected to clusters dominated by the former supervisor. This pattern has not changed over time, indicating the strong enduring similarity between their respective research lines.

**Fig 4:**
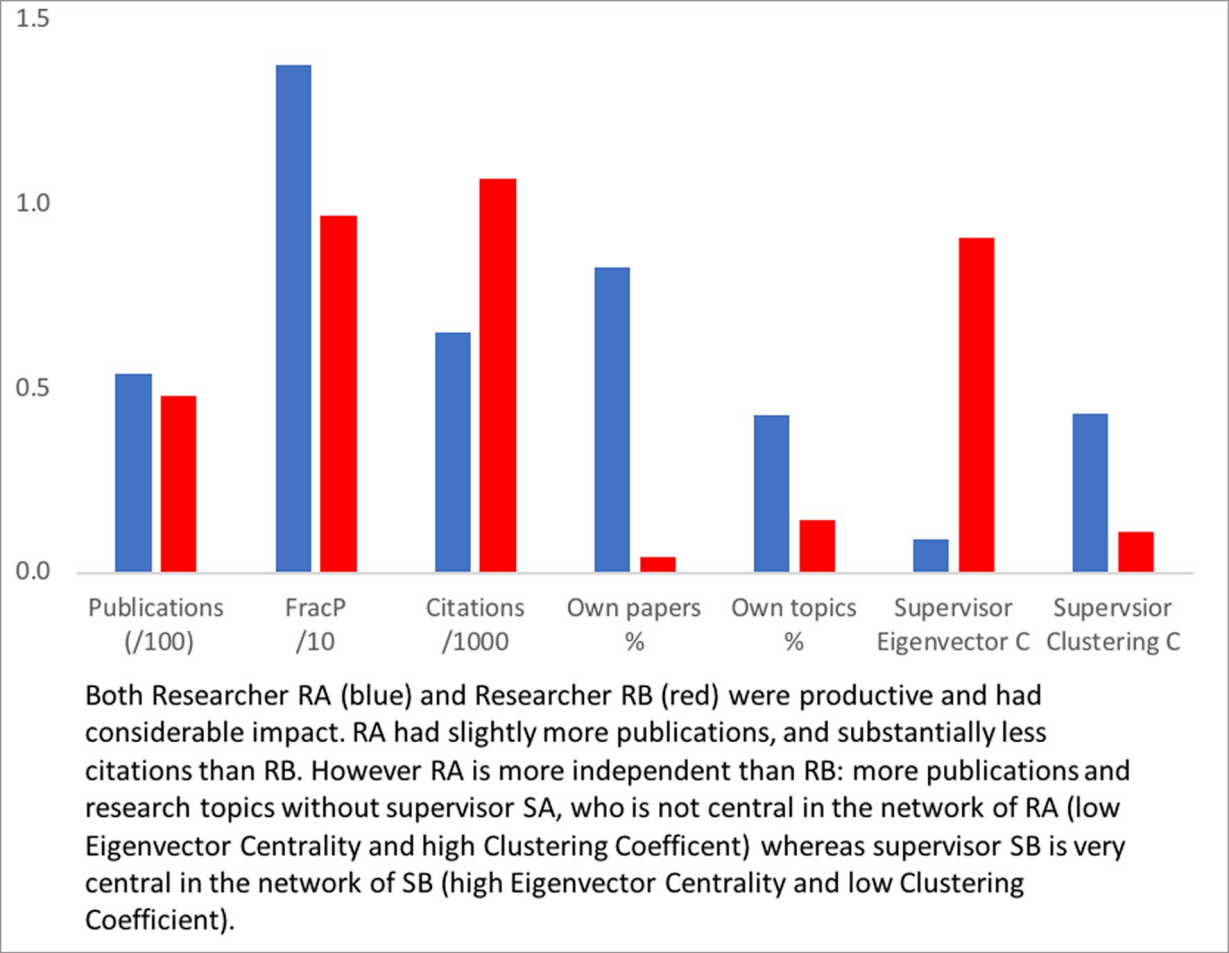
Indicator values for RA (blue) and RB (red).

So, unlike RA, RB has hardly any work outside of the large network with SB: only 4% of the papers and 14% of the topics are without SB. RB, although productive and highly cited, did not develop new research lines but remained close to the work done with supervisor SB. Concluding, two similarly talented researchers in the same field and with about the same number of co-authors, productivity and impact, show strongly different patterns in their relation to the supervisor, and this is reflected in the scores for the proposed indicators. Fig 4 summarizes the findings.

### Pair 2

As we showed in Fig 1, both researchers RC and RD were rather productive and RD had a higher citation impact. However, they co-author with their supervisors in quite a different manner: researcher RD has much more intensively co-authored with the two supervisors (75% of the papers) than researcher RC (17%). The eigenvector centrality of SC in the ego-network of RC is very low (0.04) and the clustering-coefficient of SC is very high (0.76), showing that for researcher RC the role of the supervisor in the research network is very modest. For Researcher RD, the pattern is more or less the opposite. Supervisor SD has a higher eigenvector centrality (0.28) than SC, showing that supervisor SD is more central in the ego-network of former PhD-student RD. The clustering coefficient of SD is also fairly high (0.36), and that seems to contradict with the high eigenvector centrality. However, both indicators are influenced by the specific collaboration pattern of RD who had two supervisors, who never co-authored a paper. The latter lowers the eigenvector centrality and increases the clustering coefficient of the main supervisor. In this case one may 'merge' the two supervisors, to get a better indicator for the position of the supervisors in the network of RD.

**Fig 5:**
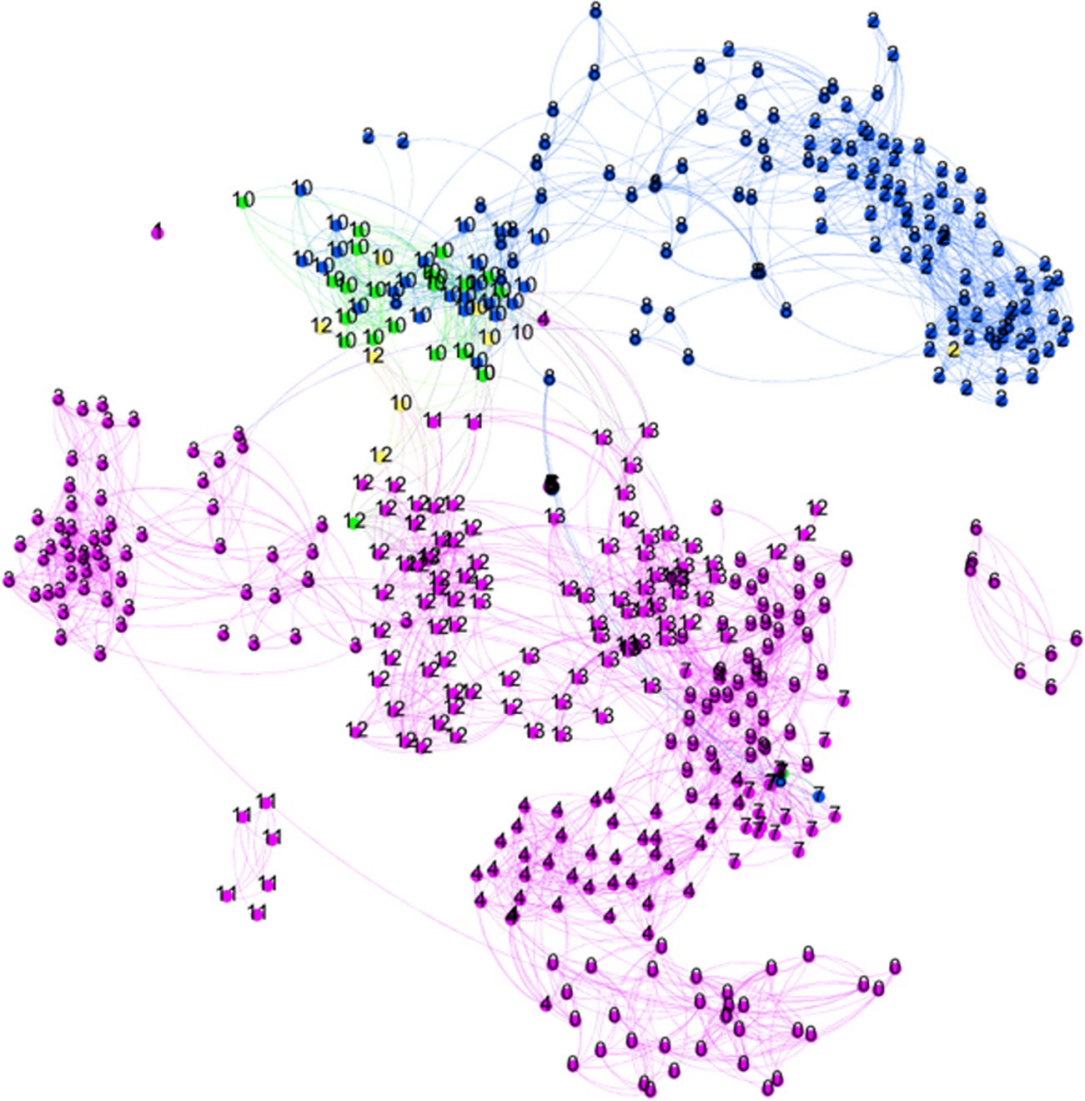
The topic network of researcher RD and the supervisors. (Legend: Blue/purple = supervisors; green = coauthored between RD and one of the supervisors; yellow = RD) Numbers indicate the topical clusters.

Is this collaboration pattern reflected in the research lines of the two researchers? As the different numbers of co-authored papers suggest, also the number of *own topics* of the researcher may differ. This is indeed the case: A large share of RC’s papers (83%) are not co-authored with the supervisor SC, and consequently, half of the topics (50%) RC is active in are not covered by the supervisor. RD, on the other hand, has no research topics without one of the two supervisors (Fig 5).

It is interesting to have a deeper look at RD: despite having 25% of the papers not co-authored with the two supervisors, these papers are in fact all in one of the research topics in which also the supervisors are active in (T10 and T12). This confirms that we indeed need more sophisticated indicators for independence than “some papers without the former supervisor” as e.g., the ERC formulates for the starting grant.

**Fig 6:**
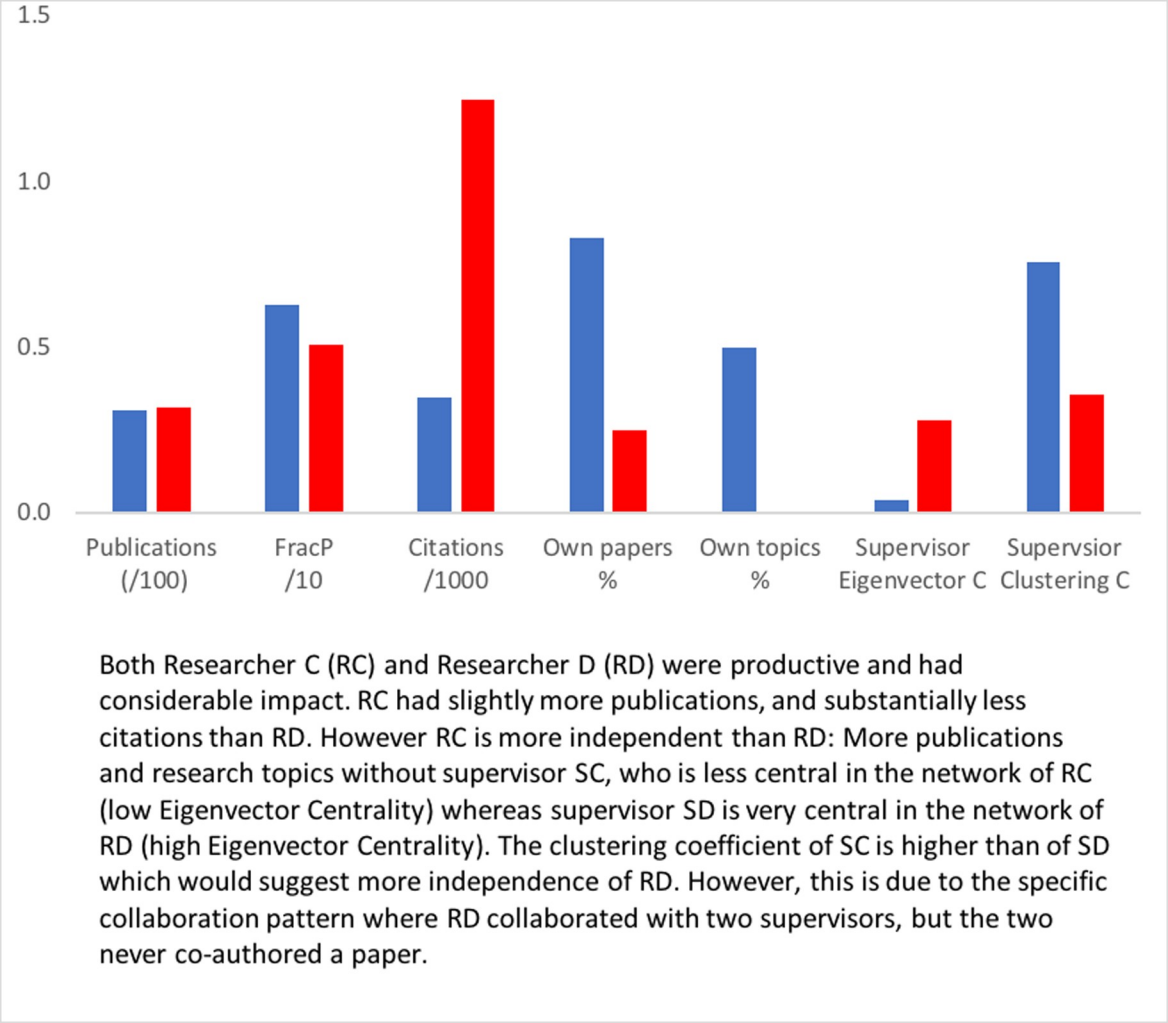
Indicator values for RC (blue) and RD (red).

RD is also interesting from another perspective. RD has two supervisors, who not co-authored any paper. At the same time, we see RD modestly authoring and co-authoring with supervisor SD1 in topic T12, and frequent authoring and co-authoring with supervisor SD2 in topic T10 (Fig 5). Given this pattern, an alternative interpretation is that RD is doing independent and original (interdisciplinary) work, linking the research lines of the two supervisors. Which of the interpretations is correct, depends on the moment of publishing: were RD’s own papers in the two clusters published earlier than those of the supervisors or the other way around? Inspecting the data suggests dependency, as the supervisors started to publish in those topics years before RD entered them. Fig 6 summarizes the findings.

## Conclusions and discussion

This paper started by defining quality as a more-dimensional construct and argued that there is a need for extending the indicators portfolio reflecting this multi-dimensionality. Then we argued that independence is an important dimension, which is emphasized in a number of studies and reports. We proposed four indicators for this: two for the position of the former supervisor in the co-author network of an early career researcher, and two for the position of the former supervisor in the topic network of the early career researcher. The four indicators give a rather consistent picture: each of them places the researchers in about the same ranking, and together they may constitute a valid overall indicator of independence. The overall indicator scores clearly distinguish RA and RC from RB and RD (Table 3). When using the granularity level 0.5 or 1.0, the overall indicator gets the values 0.89, 0.79, 0.33, and 0.19 –only marginally different from the granularity level 1.5 as reported in Table 3.

**Table 3:** Independence indicator.

Rank order	Researcher		Overall score	Centrality Supervisor (i)	Clustering Supervisor (ii)	Own papers (iii)	Own topics* (iv)
1. Most independent		RC	0.89	0.04	0.76	0.83	0.50
2.		RA**	0.76	0.09	0.43	0.83	0.43
3.		RD***	0.33	0.28	0.36	0.25	0.00
4. Least independent		RB	0.13	0.91	0.11	0.04	0.14

* Resolution level = 1.5

** We use here the first supervisor for calculating the indicators; the second supervisor gives similar values.

*** We use the second supervisor, as this supervisor was the main collaborator of RD (Fig 5).

Interestingly, RA had an equal output and a lower impact than RB, and RC had a somewhat lower output and a much lower impact than RD. But RA and RC had the successful academic career whereas the others stopped, indicating that the commonly discussed publication-based and citation-based indicators may not be really informative. At this career stage, one assumes rather good numbers of publications (productivity) and citations (impact), and differences between those may not decisive, whereas other evaluation dimensions may play a role. In our cases, the career success does correspond with the independence scores. We do not suggest that independence is always the decisive variable, as job selection is a multi-criteria decision-making problem. But it is at least one of the more important quality dimensions.

In this era of *team science*, it is not anymore possible to show independence by single authored papers, therefore there is a need for more sophisticated assessment methods, and our proposal in this paper seems to be a step in that direction. Further testing and further development are needed, and several issues need to be addressed in order to improve the indicator:

- The researchers we used as examples all came from STEM fields; using the WoS data then seems a reasonable approach. However, as is often argued, for other fields the WoS is not covering enough of the output. Partly this is a statistical issue: If the WoS papers are representative for the whole oeuvre, also in terms of co-authorships, then relying on the WoS data may not be problematic. Nevertheless, we would agree that the indicators should be tested on other datasets too. This was done for a social science case and a computer science case [42]–two fields that have publication patterns that do not strongly focus on journal articles. Using Google Scholar data, the study suggests that the proposed independence indicator can be generalized to other data sources than only bibliometric databases as WoS or Scopus.
- The independence indicator is based on a model of science which assumes research fronts, where researchers build on (and cite) earlier work in order to contribute to the growth of knowledge. It is regularly argued that this model does not fit on all fields, as the concept of knowledge growth does not apply to all disciplines. Especially fields within the social sciences and even more in the humanities have different goals, such as providing meaning. The main audience in such fields may not be scholars, but other audiences–such as the general public. The differences in the intellectual and social organization of the disciplines [43] may lead to different sources of reputation and different ways of assessing performance (and reputation control). In line with this, various efforts have been made to develop discipline-based performance indicators–often by interviewing the researchers what they consider quality and how they would prefer to be evaluated [19, 44, 45], or by analyzing how elements of quality (such as originality) are differently perceived between the disciplines [46]. On the other hand, ‘epistemological cultures’ may change over time, and recent research suggests that publishing habits between the various disciplines are converging [47], which suggests that bibliometric indicators can be used in more and more research fields.
- Another extension would be to introduce a temporal dimension–one could imagine that an early career researcher starts a new topic without the supervisor(s) being involved, with the supervisors later jumping on that bandwagon when it proves to be successful. Then a joint topic is not proof of dependence–on the contrary. This phenomenon was discussed for researcher D and the test we did should be included when using the proposed indicators.
- Similarly, dependency may not only occur with the previous supervisor, but also with other collaborators in the early career. To control for that, the indicators should probably be calculated for all frequent co-authors of the early career researcher.
- For individual evaluations, data cleaning and calculations can be easily done. However, it would be useful to test on a large sample whether the independence indicator predicts success better than other variables. In such a case data collection and cleaning may be resource and time intensive. That type of procedure indeed should be done for all indicators: their real-life value should be shown in a multivariate prediction of career or grant success. If the independence indicators as defined here have validity, such test would show whether independence does make a difference.

Overall, this paper illustrates how to derive more indicators for different quality dimensions of scholarly work. We would emphasize that this is the better way to move forward: if current indicators are not adequate (enough) as claimed e.g., in the Leiden Manifesto [8], one should try to develop better indicators, and not reduce the role of indicators and reinforce the role of peer review. Peer review is more problematic than indicators–as we argued in the introduction. Interestingly, the tendency in many debates seems to be that less use of indicators is the better alternative for not so good indicators. Here we follow a different strategy: if current indicators are not covering the various quality dimensions and lack validity, let’s develop better indicators for those dimensions of quality not yet addressed. This seems more relevant than trying to marginally improve the existing ones [48].

## Supporting information

S1 Dataset Collaboration data. Data files used to calculate ego network measures.
(XLSX)

S2 Dataset Topic data. Data files used to calculate the share of independent research topics.
(XLSX)

## References

[1] Wildgaard L, Schneider JW, Larsen B (2014). A review of the characteristics of 108 author-level bibliometric indicators. *Scientometrics* 101:1–158.

[2] Sandström E & Sandström U (2009). Meeting the micro-level challenges: Bibliometrics at the individual level. *Proceedings ISSI* 2009.

[3] Costas TN, Van Leeuwen T, Bordons M (2010). A bibliometric classificatory approach for the study and assessment of research performance at the individual level: The effects of age on productivity and impact. *Journal of the American Society for Information Science and Technology* 61 (8): 1564–1581

[4] Bornmann L & Marx W (2014a). Distributions Instead of Single Numbers: Percentiles and Beam Plots for the Assessment of Single Researchers. *Journal of the Association for Information Science and Technology* 65(1):206–208. 10.1002/asi.22996

[5] Bornmann L & Marx W (2014b). How to evaluate individual researchers working in the natural and life sciences meaningfully? A proposal of methods based on percentiles of citations. *Scientometrics* 98:487–509.

[6] Bollen J, Van de Sompel H, Hagberg A & Chute R (2009) A Principal Component Analysis of 39 Scientific Impact Measures. *PLoS ONE* 4(6): e6022 10.1371/journal.pone.0006022 19562078 PMC2699100

[7] Yan E & Ding Y (2011). Discovering author impact: A PageRank perspective. *Information processing and management*, 47(1), 125–134.

[8] Hicks D, Wouters P, Waltman P, de Rijcke S, Rafols I (2015). The Leiden Manifesto for research metrics. *Nature* 2015:429–431 (23 April 2015) 10.1038/520429a . 25903611

[9] Wilsdon J, Allen L, Belfiore E, Campbell P, Curry S, Hill S, et al (2015). *The Metric Tide*: *Report of the Independent Review of the Role of Metrics in Research Assessment and Management*. Report 2015. 10.13140/RG.2.1.4929.1363

[10] Dijstelbloem H, Huisman F, Miedema F & Mijnhardt W (2013). Why Science Does Not Work as It Should and What To Do about It. *Science in Transition* *Position Paper*–October 17, 2013. (http://www.scienceintransition.nl/wp-content/uploads/2013/10/Science-in-Transition-Position-Paper-final.pdf retrieved 2017-09-29).

[11] Van Raan A (2005). Fatal Attraction: Conceptual and methodological problems in the ranking of universities by bibliometric methods. *Scientometrics* 62 (1): 133–143.

[12] Chubin DE and Hackett EJ (1990). Peerless science: peer review and U.S. science policy. Albany: State Univ of New York Press.

[13] Sandström U & van den Besselaar P (2016). Quantity and/or Quality? The Importance of Publishing Many Papers. *PLoS ONE* 11(11): e0166149 10.1371/journal.pone.0166149 27870854 PMC5117611

[14] Van den Besselaar P, Heyman U, Sandström U (2017). Perverse effects of output-based research funding? Butler’s Australian case revisited. *Journal of Informetrics* 11, 905–918.

[15] Narin F (1976). Evaluative bibliometrics: The use of publication and citation analysis in the evaluation of scientific activity New Jersey: Computer Horizons Inc.

[16] Vieira ES, Cabral JAS & Gomes JANF (2014). How good is a model based on bibliometric indicators in predicting the final decisions made by peers? *Journal of Informetrics* 8: 390–405.

[17] Gulbrandsen M (2000). Research quality and organizational factors: an investigation of the relationship. (PhD dissertation) NTNU: Trondheim.

[18] Moher D, Naudet F, Cristea IA, Miedema F, Ioannidis JPA, Goodman SN (2018). Assessing scientists for hiring, promotion, and tenure. *PLoS Biol* 16(3): e2004089 10.1371/journal.pbio.2004089 29596415 PMC5892914

[19] Hemlin S (1993). Scientific quality in the eyes of the scientist: a questionnaire study. Scientometrics 27 (1): 3–18.

[20] Van Arensbergen P, Van der Weijden I, Van den Besselaar P (2014). Different views on scholarly talent–what are the talents we are looking for in science? *Research Evaluation* 23, 273–284.

[21] Bozeman B, Youtie J (2018). *The strength in numbers*: *the new science of team science*. Princeton University Press.

[22] Henriksen D (2016). The rise in co-authorship in the social sciences (1980–2013). Scientometrics 107 (2): 455–476.

[23] Wagner CS, Park HW, Leydesdorff L (2015). The Continuing Growth of Global Cooperation Networks in Research: A Conundrum for National Governments. *PLoS ONE* 10 (7): e0131816 10.1371/journal.pone.0131816 26196296 PMC4510583

[24] Van den Besselaar P, Inzelt A-M, Reale E, de Turckheim E, Vercesi V (2012). Indicators for internationalization of research institutions. Strasbourg: European Science Foundation.

[25] NAS (2005). Bridges to Independence: Fostering the Independence of New Investigators in Biomedical Research. Washington (DC): National Academy Press. 20669450

[26] Cech TC (2005). Fostering Innovation and Discovery in Biomedical Research. *JAMA* 294(11):1390–1393. 10.1001/jama.294.11.1390 16174699

[27] Neufeld J, Huber N & Wegner A (2013). Peer review-based selection decisions in individual research funding, applicants' publication strategies and performance: The case of the ERC Starting Grants. *Research Evaluation* 22 (4): 237–247.

[28] Thomas D & Nedeva M (2012). Characterizing researchers to study research funding agency impacts: The case of the European Research Council's Starting Grants. *Research Evaluation* 21 (4): 257–269.

[29] Mongeon P, Smith E, Joyal B, Lariviere V, (2017). The rise of the middle author: Investigating collaboration and division of labor in biomedical research using partial alphabetical authorship. *PlosOne* 12, 9, 0184601 10.1371/journal.pone.0184601 PMC5599011 28910344

[30] Collins H (2010). Knowing what we don't know. *New Scientist* 206, Issue 2762.

[31] Working group for quality in research: Proposals for improved assessment criteria for applications. Report from a working group at the The Scientific Council for Medicine and Health, October 2007. Chair: Stefan Lohmander. (In Swedish).

[32] Wennerås C & Wold A (1997). Nepotism and sexism in peer-review. *Nature* 387, 341–343 (22 May 1997), 10.1038/387341a0 9163412

[33] Ahlqvist V, Andersson J, Hahn Berg C, Kolm C, Söderqvist L & Tumpane J (2013). *Observations on gender equality in a selection of the Swedish Research Councils’ evaluation panels*. Vetenskapsrådet Report, ISBN 978-91-7307-224-3.

[34] Horlesberger M, Roche I, Besagni D, Scherngell T, Francois C, Cuxac P, et al, (2013). A concept for inferring 'frontier research' in grant proposals. *Research Evaluation* 22 (2): 129–148.

[35] Wang Q (2018). A Bibliometric Model for Identifying Emerging Research Topics. *Journal of the association for information science and technology*, 69(2):290–304

[36] Uzzy B, Mukherjee S, Stringer M, Jones B (2013), A-typical combinations and scientific impact. *Science* 342, Issue 6157, pp. 468–472. 10.1126/science.1240474 24159044

[37] Wang J, Veugelers R, Stephan P (2017). Bias against novelty in science: A cautionary tale for users of bibliometric indicators. *Research Policy* 46(8), 1416–1436.

[38] Gazni A, Sugimoto C, Didegah F (2012). Mapping world scientific collaboration: authors, institutions, and countries. *JASIST* 63, 323–335.

[39] Salton G, Mcgill M (1983). *Introduction to Modern Information Retrieval*. New York: McGraw-Hill.

[40] Waltman L & Eck NJ (2013). A smart local moving algorithm for large-scale modularity-based community detection. *European Physical Journal B* 86 (11): 471 10.1140/epjb/e2013-40829-0

[41] Van Balen B, van Arensbergen P, van der Weijden I & van den Besselaar P (2012). Determinants of Success in Academic Careers. *Higher Education Policy* 25, 313–334

[42] Dumitrache A, Groth P, van den Besselaar P (2013). Identifying research talent using web-centric databases. In: Davis H, Halpin H, Pentland A, Bernstein M, Adamic L, Alani H, Monnin A, Rogers R (eds), *Proc*. 3rd Annual ACM Web Science Conference, Paris, pp 57–60.

[43] Whitley R (2000 (1984)). The intellectual and social organization of the sciences Second Edition Oxford: Oxford Univ Press.

[44] Hemlin S & Gustafsson M (1996). Research production in the Arts and Humanities: a questionnaire study of factors influencing research performance. *Scientometrics* 37 (3): 417–432.

[45] Hug SE, Ochsner M & Daniel HD (2013). Criteria for assessing research quality in the humanities: a Delphi study among scholars of English literature, German literature and art history. *Research Evaluation*, 22(5), 369–383.

[46] Barlösius E (2019) Concepts of Originality in the Natural Science, Medical, and Engineering Disciplines: An Analysis of Research Proposals. *Science*, *Technology & Human Values* (Online first).

[47] Bonaccorsi A, Darario C, Fantoni S, Folli V, Leonetti M & Roucco G (2107). Do social sciences and humanities behave like life and hard sciences? Scientometrics 112: 607–653.

[48] Osterloh M, Frey BS (2015). Research governance in academia: are there alternatives to academic rankings? In Cronin B & Sugimote CR eds. *Scholarly metrics under the microscope*. ASIST 2015.

[49] Van den Besselaar P, Sandström U, Van der Weijden I (2002). The independence indicator Archambault E., Gingras Y., Lariviere V. (eds.) *Science & Technology Indicators* *2012* Montreal: OST & Science Metrix, 131–141

